# Maternal obesity and programming of metabolic syndrome in the offspring: searching for mechanisms in the adipocyte progenitor pool

**DOI:** 10.1186/s12916-023-02730-z

**Published:** 2023-02-13

**Authors:** Taylor B. Scheidl, Amy L. Brightwell, Sarah H. Easson, Jennifer A. Thompson

**Affiliations:** 1 Cumming School of Medicine, Calgary, Canada; 2 Alberta Children’s Hospital Research Institute, Calgary, Canada; 3 Libin Cardiovascular Institute, Calgary, Canada; 4 University of Calgary, 3330 Hospital Dr. NW, Calgary, AB T2N 4N1 Canada

**Keywords:** Obesity, Gestational diabetes, Metabolic syndrome, Fetal programming, Adipose tissue

## Abstract

**Background:**

It is now understood that it is the quality rather than the absolute amount of adipose tissue that confers risk for obesity-associated disease. Adipose-derived stem cells give rise to adipocytes during the developmental establishment of adipose depots. In adult depots, a reservoir of progenitors serves to replace adipocytes that have reached their lifespan and for recruitment to increase lipid buffering capacity under conditions of positive energy balance.

**Main:**

The adipose tissue expandability hypothesis posits that a failure in de novo differentiation of adipocytes limits lipid storage capacity and leads to spillover of lipids into the circulation, precipitating the onset of obesity-associated disease. Since adipose progenitors are specified to their fate during late fetal life, perturbations in the intrauterine environment may influence the rapid expansion of adipose depots that occurs in childhood or progenitor function in established adult depots. Neonates born to mothers with obesity or diabetes during pregnancy tend to have excessive adiposity at birth and are at increased risk for childhood adiposity and cardiometabolic disease.

**Conclusion:**

In this narrative review, we synthesize current knowledge in the fields of obesity and developmental biology together with literature from the field of the developmental origins of health and disease (DOHaD) to put forth the hypothesis that the intrauterine milieu of pregnancies complicated by maternal metabolic disease disturbs adipogenesis in the fetus, thereby accelerating the trajectory of adipose expansion in early postnatal life and predisposing to impaired adipose plasticity.

## Background

It is well established that maternal metabolic disease during pregnancy predicts the development of obesity and cardiometabolic disease in the offspring. Research over the past two decades in the obesity field has shed light on the central role of adipose tissue remodeling in the maintenance of systemic metabolic homeostasis. The ability of adipose tissue to adapt to an obesogenic environment by sequestering and buffering toxic lipids is dependent on a resident pool of adipocyte progenitors that can be recruited for differentiation. Mouse models of lineage tracing and adipocyte labeling have identified the late fetal period as a critical window that establishes the adipocyte progenitor pool and trajectory of fat accumulation in early postnatal life. These seminal studies highlight the in utero environment as a critical regulator of postnatal adiposity and adipose plasticity; however, the influence of maternal metabolic health on progenitor populations in adipose tissue of the offspring remains largely unexplored. Since the primary outcome of pregnancies complicated by maternal obesity or diabetes is excessive neonatal adiposity, it is likely that later life cardiometabolic disease stems from a perturbation in adipose development. In this narrative review, we describe current knowledge with respect to the developmental time course of lineage commitment and differentiation of adipose progenitors and their role in the pathophysiology of cardiometabolic disease. Later, we summarize the literature to-date supporting a role for aberrant fetal adipogenesis as a primary mechanism responsible for programming of cardiometabolic disease in offspring born to pregnancies complicated by maternal obesity or diabetes.

### The obesity crisis

The prevalence of obesity in Western nations was 13% in the late twentieth century [1], climbing to 35% over the last two decades. Today, obesity is one of the most significant public health problems, affecting 650 million individuals globally [2], a crisis driven by multifaceted environmental, behavioral, and socioeconomic factors. Socioeconomic disparities in obesity burden exist within high income countries, while globalization of the food and beverage industry has led to a double burden of obesity and malnutrition in the global south. Building more resilient populations and mitigating the burgeoning rise in health care costs are incumbent upon addressing the obesity crisis.

### Obesity in childhood and the reproductive years

The age-of-onset of obesity has declined in parallel with its increasing prevalence. Over the past several decades, the rise in obesity has occurred more rapidly in children and adolescents than in adults. A study published in the *Lancet* showed that between 1975 and 2016, obesity among children and adolescents increased globally, with an increase of 30–50% per decade in high income countries and an increase of 400% in parts of Africa [3]. More recently, obesity rates among youth appear to have plateaued [4, 5], albeit at high levels (18–20%) in some high-income regions including North America [6, 7]. Currently, it is unclear how future trends of childhood obesity will be impacted by the COVID-19 pandemic, which resulted in a doubling of the rates of BMI increase in children, with the most drastic changes observed in 5–11-year-olds [8, 9]. Obesity at younger ages increases the duration over which the obesity burden is carried throughout the life course. A meta-analysis published by Simmonds et al. reported that 55% of obese children become obese as adolescents, while 80% of adolescents that are obese remain obese in adulthood [10]. Data collected in the USA show that obesity at younger ages increases years of lost life across the lifespan [11]. Earlier onset of obesity-associated morbidity due to higher rates of childhood obesity will come with high economic costs for future generations with impacts to productivity in the working age population and greater burden on health care systems.

Consequent to the declining age-of-onset, a greater number of women are overweight or obese at reproductive age. In 2019, 29% of US women were obese immediately prior to pregnancy, an increase of 11% since 2016 [12]. Maternal obesity has become one of the most common complications of pregnancy and is a major risk factor for gestational diabetes (GDM), both of which increase the risk for adverse perinatal outcomes and later-life chronic disease [13–16]. Mothers that are obese or diabetic during pregnancy are more likely to have babies that develop obesity, often before adulthood [17–19]. Therefore, this transmission of obesity across generations may be amplifying its prevalence and contributing to its declining age of onset.

### Obesity and the metabolic syndrome

Obesity is a central pathophysiological mechanism in development of the metabolic syndrome (MetS), a clustering of risk factors for cardiovascular disease (CVD) including dyslipidemia, hypertension, and insulin resistance [20, 21]. According to NHANES data (2011–2016), 20% of 20–34-year-olds and 49% of individuals over 60 in the USA have MetS [22]. The presence of MetS doubles the risk for CVD and increases the risk for T2DM by 6-fold [23]. Results from the Global Burden of Disease study showed CVD to account for 41% of deaths and 34% of disability-adjusted life years related to high BMI [24]. Among adults with T2DM, more than 80% of individuals were overweight or obese [25], suggesting that the prevalence of T2DM is largely driven by obesity. Therefore, obesity and MetS are major drivers of CVD and T2DM, two main causes of mortality and morbidity in populations across the globe.

The childhood obesity crisis has resulted in an earlier onset of MetS. Among children and adolescents, the prevalence of MetS is reported to fall between 2.5 and 3.5% in Canada and between 4.2 and 9.2% in the USA [26]. At least one component of MetS is present in one third of youth in North America [26], with abdominal obesity being the most common. According to NHANES data (2015-2018), obesity is a common co-morbidity in children and adolescents with hypertension, which occurred in 4.6% of children between the ages of 8–12 and in 3.7% of adolescents between the ages of 13–17 [27]. This trend of obesity-associated cardiometabolic disorders at younger ages is predicted to reverse the steady rise in life expectancy that has occurred over the past two centuries, as the burden of morbidity will span further across the life course in future generations [28]. Given the rise in obesity at reproductive age and associated metabolic complications of pregnancy, it is plausible that transmission of obesity across generations due to fetal overnutrition has played a key role in the emergence of pediatric MetS. Several studies have shown that development of obesity and MetS before adulthood is more common in babies born to mothers with metabolic disease during pregnancy [14–16]. Dabelea et al. showed that over a 10-year period, the increase in prevalence of T2DM in Pima children was almost entirely accounted for by exposure to maternal diabetes during pregnancy [29]. Therefore, preventative measures targeted to metabolic health during pregnancy are critical in efforts to mitigate the global epidemic of MetS and its declining age of onset.

### The central role of adipose tissue

Obesity is characterized by the excessive accumulation of white adipose tissue (WAT), which is distributed throughout the body in anatomically distinct depots that fulfill related but unique functional roles [30]. Regardless of anatomic location, the principal role of WAT is to serve as a “metabolic sink” that stores energy in the form of triglycerides packaged in lipid droplets of adipocytes, the functional units of WAT. Additionally, WAT synthesizes and secretes adipokines that play a key role in the regulation of systemic metabolism through paracrine and endocrine actions. The first adipokine to be discovered was leptin, which is produced by adipocytes and functions in the regulation of hunger and satiety through crosstalk with neuronal targets [31]. Acting on its cognate receptors in the hypothalamus, leptin stimulates hunger in the fasted state and dampens it in the fed state. Adiponectin is specifically expressed and secreted from adipocytes, predominately in subcutaneous (SAT) depots, and enters the circulation to act distally on the liver and muscle where it enhances sensitivity to insulin, increases fatty acid oxidation, and decreases influx of fatty acids [32]. Thus, WAT orchestrates responses to nutritional and environmental cues for fine control of systemic lipid metabolism and energy balance

### Body fat distribution, not absolute fat mass, confers risk

The primary anatomical divisions of WAT are the subcutaneous (SAT) and visceral adipose tissue (VAT) depots. The majority of WAT volume is localized to the SAT [33], located just beneath the skin and is the principal site of energy storage. The SAT is considered a protective adipose depot as its expansion is associated with a favorable metabolic phenotype [30]. The VAT, located primarily in the abdominal cavity surrounding the organs, comprises only 10–20% and 5–8% of total body fat in men and women, respectively [33]. Accumulation of fat in the abdominal region and VAT compartments is a pattern of obesity termed central or android obesity that is associated with higher cardiometabolic risk and is characteristic of males [34]. Waist-to-hip circumference, a proxy measurement of central obesity, is a better predictor of T2DM and CVD than BMI [35, 36]. The protection of females from cardiometabolic disease relative to males during the reproductive years is at least in part attributable to their tendency to accumulate fat in the gluteo-femoral region and SAT depots. This protection is lost after menopause when the distribution of body fat shifts to a male-like pattern with higher expansion of VAT [37–39]. The favorable influence of SAT on metabolic homeostasis has been experimentally demonstrated by the improvement in insulin sensitivity, plasma lipid profiles, and hepatic lipid content after autologous transplantation of SAT into VAT depots in rodent models of diet-induced obesity [40, 41]. Overall, there is abundant evidence showing that distribution across anatomical depots, rather than total body fat mass, is the important factor driving the relationship between obesity and risk for MetS.

### The adipose progenitor pool is recruited to expand lipid storage capacity

Under obesogenic conditions, WAT expands to accommodate the energy surplus by two mechanisms: hypertrophy of existing adipocytes and hyperplastic expansion in which new adipocytes are generated by recruiting a resident population of adipose-derived stem cells (ASC) for differentiation. Adipogenesis generates new adipocytes through sequential differentiation steps, in which multipotent mesenchymal stem cells commit to the adipocyte lineage and subsequently become preadipocytes, followed by terminal differentiation to form a mature lipid-storing adipocyte. While the transcriptional program that controls lineage specification and differentiation is well-defined, much remains to be learned with respect to environmental cues that govern the behavior of progenitors within their native microenvironment.

It is thought that proliferation and differentiation of ASC are stimulated through paracrine feedback when hypertrophic growth has reached a defined limit of capacity. Pro-adipogenic signals released from engorged adipocytes may be triggered by mechanical strain [42], local hypoxia [43], or hypertrophy-induced cell death [44]. Recently, Li et al. demonstrated that adipocytes from obese or insulin-resistant humans were prone to undergoing premature senescence [45], raising the possibility of a mechanism common to adipocytes that have either reached a critical size or the end of their lifespan. While the signals emanating from adipose tissue to recruit the ASC pool for differentiation remain unresolved, current evidence demonstrates that adipose expansion is associated with a shift in the adipose secretome towards higher release of pro-inflammatory mediators [46–48]. Interestingly, a recent study showed an increase in differentiation of ASC exposed to the secretome of adipose explants from high fat-fed mice [46]. Adipocyte-specific dominant-negative mutation of the pro-inflammatory cytokine, TNFα, prevented diet-induced WAT expansion, demonstrating that adipocyte inflammation is required for adaptive WAT remodeling [49]. It is also possible that non-adipocyte cells within the adipose microenvironment mediate crosstalk with the ASC pool. Indeed, a number of studies demonstrate that the pro-inflammatory environment of expanding adipose tissue is in part attributable to macrophages that surround and eliminate dead adipocytes [49]. Work by Lee et al. demonstrated that macrophages recruited to dead adipocytes secret a chemotactic signal that activates local progenitors [50]. The above evidence suggests that the ASC pool senses and responds to a local and transient inflammatory environment, contributing to adaptive adipose tissue remodeling that replaces dead or senescent adipocytes. Redox signaling plays a key role in mediating cellular responses to inflammation and recent work from our laboratory reveals a novel role for reactive oxygen species (ROS) in stimulating adipogenesis [51]. Thus, ROS may serve as second messengers in the transduction of signals from the local environment to control behavior of the progenitor pool. New techniques that preserve cell-to-cell and cell-to-matrix interactions within the adipose microenvironment will shed light on signaling pathways that govern the response of ASC to the energy buffering needs of WAT.

### Adipogenic responses in obesity are depot- and sex-specific

Recruitment of the progenitor pool allows WAT depots to meet increased requirements for lipid storage in the face of a prolonged positive energy balance. The adipogenic response to lipid overload is depot specific. Traditional thought held that stimulation of adipogenesis in response to an obesogenic diet occurs primarily in the SAT. This supposition was based on studies measuring adipogenic potential in ASC isolated from different adipose depots, cultured, and exposed to a differentiation cocktail in vitro. This experimental approach consistently demonstrates higher intrinsic adipogenic potential in ASC isolated from SAT compared to those isolated from VAT [52, 53]. Further, a study by Tchoukalova et al. revealed lower adipogenic capacity in ASC isolated from abdominal SAT compared to those isolated from the femoral depot, suggesting variability across subcutaneous depots [54]. However, newer in vivo approaches such as pulse-chase transgenic lineage tracing have called into question the assumption that intrinsic adipogenic potential in vitro reflects adipogenic responses of ASC in their native environment. In mouse models of high fat feeding, proliferation of ASC occurs rapidly in the first 12 h in both VAT and SAT but thereafter is observed in only VAT [55]. By 8 weeks, proliferating ASC undergo differentiation in VAT, as indicated by an increase in BrdU incorporation into mature adipocytes and decrease in proliferating ASC [55]. Using a pulse-chase system of labeling pre-existing adipocytes, Wang et al. demonstrated that after 35 days of high fat feeding, adipocyte hypertrophy was apparent in both SAT and epididymal VAT, while after 56 days of the obesogenic diet new adipocytes were generated in epididymal fat only [56]. Likewise, another model of inducible adipocyte tagging that distinguishes new from pre-existing adipocytes showed that diet-induced adipogenesis occurred predominately in VAT [55]. Interestingly, the same group subsequently published a study revealing that diet-induced adipogenesis exclusively in VAT is a male-specific response. In females, diet-induced proliferation and differentiation of ASC occurred in both SAT and VAT, leading to a similar increase in mass of these two depots [57]. Ovariectomy in female mice recapitulated the VAT-dominant adipogenic response observed in males [57]. The default exclusion of females in pre-clinical studies has obscured our understanding of how sex influences WAT responses in obesity. Sexual dimorphism in behavior of the adipocyte progenitor pool may be a key contributor to sex differences in patterns of body fat distribution and risk for cardiometabolic disease.

Gene expression signatures and differentiation potential of ASC vary across adipose depots, suggesting that cell-autonomous properties contribute to depot-specific responses to lipid storage needs [53]. A role for the microenvironment in regulating the adipocyte progenitor pool has also been demonstrated. While low adipogenic potential is characteristic of ASC isolated from VAT and cultured in a monolayer [53], adipogenic capacity was similar between VAT and SAT-derived ASC differentiated in 3D culture that recapitulates the cell-to-cell and cell-to-matrix interactions of the adipose microenvironment [58]. Jeffrey et al. found that ASC isolated from SAT of male mice differentiate readily when transplanted in VAT of male recipients exposed to an obesogenic stimulus, while the adipogenic response was lower when ASC were isolated from VAT and transplanted into SAT [57]. Therefore, inhibitory or stimulatory signals originating from the microenvironment may be more important in regulating adipogenic responses than cell-intrinsic differentiation potential. Females are less susceptible to high fat diet-induced adipocyte apoptosis and inflammation [59], which occurs predominately in the VAT of obese males [60, 61], possibly explaining sex and depot-specific differences in adipogenic responses.

### The adipose tissue expandability hypothesis

**Fig. 1:**
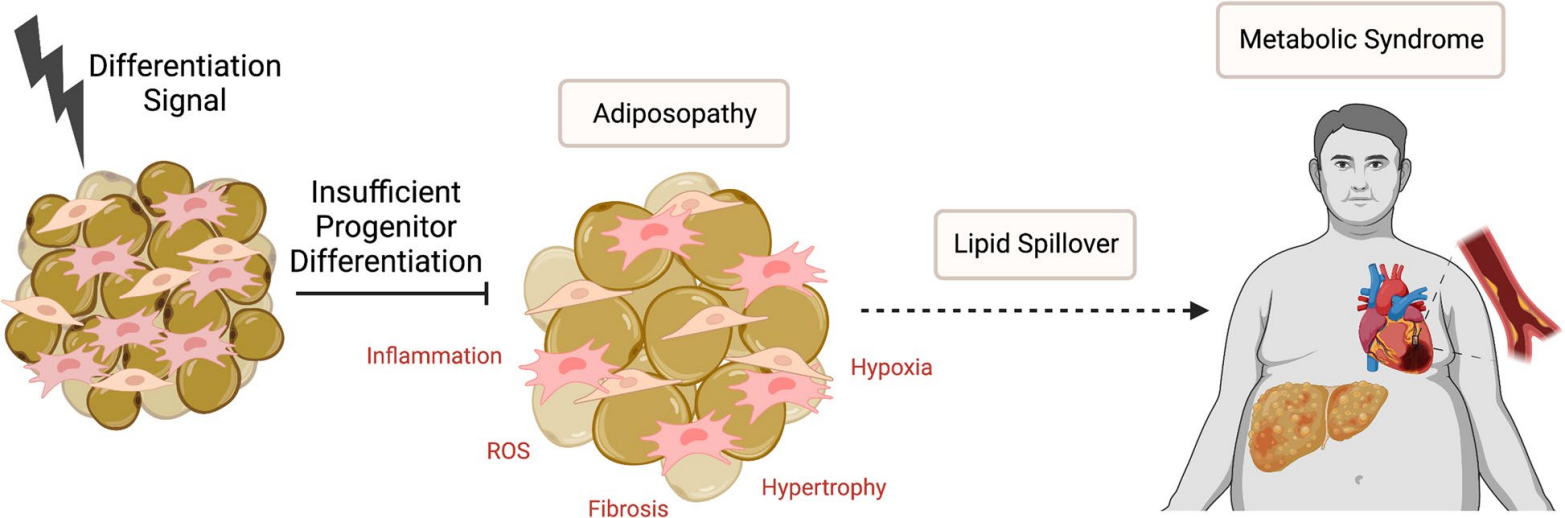
Adipose tissue expandability hypothesis. In established adult depots, a resident population of adipocyte progenitors serves to support normal cell turnover and as a reservoir to increase lipid buffering capacity under conditions of energy surplus. Impairment in the ability of progenitors to differentiation and generate new cells forces WAT expansion to rely exclusively on existing adipocytes, leading to adiposopathy. Adiposopathy is characterized by excessive adipocyte hypertrophy, fibrosis, hypoxia, inflammation, and blunted responses to the anti-lipolytic effects of insulin. Dysfunctional WAT cannot meet energy storage demands, and spills lipids into the circulation, leading to ectopic deposition in organs such as the blood vessels and livers and accumulation in secondary, VAT depots

The “adipose tissue expandability hypothesis” posits that a failure of SAT to meet energy buffering needs of the body triggers the onset of obesity-induced cardiometabolic disease (Fig. 1). An overreliance on hypertrophic growth results in engorged adipocytes that become hypoxic, fibrotic, and inflamed. Dysfunctional adipocytes in obesity-associated adiposopathy or “sick fat” lose their ability to buffer energy and become resistant to the anti-lipolytic effects of insulin, leading to lipid spillover into the circulation, increased lipid storage in visceral depots, and deposition in ectopic organs such as the liver and arteries [62, 63]. Adiposopathy is characterized by an imbalance in lipid handling with increased lipogenesis and lipolysis and decreased fatty acid oxidation, as well as dysregulated adipokine secretion. Retained expansion capacity of SAT is thought to distinguish those with unhealthy obesity from the metabolically healthy obese (MHO), a term used to describe individuals with a BMI that falls in the obese category (BMI ≥ 30 kg/m^2^) but absent of other components of MetS (e.g., insulin resistance, hypertension) [64–66]. Overexpression of adiponectin in the *ob/ob* mouse model of obesity exacerbates total weight gain, primarily as a consequence of increased SAT volume, decreases adipocyte diameter in SAT, and normalizes insulin sensitivity and hepatic lipid content [67]. Insulin sensitivity is maintained in mice that develop obesity as a consequence of collagen VI deficiency that results in unrestricted WAT expansion [68]. This study not only demonstrates the adaptive role of WAT expansion in obesity but also highlights that the extracellular scaffold imposes a barrier to WAT expansion that may play a role in impaired expandability when hypertrophic adipocytes become fibrotic. These transgenic models provide strong evidence supporting a critical role for adipose plasticity in determining metabolic health in obesity. In fact, weight gain is a common side-effect of thiazolidinediones (TZD), a class of anti-diabetic drugs that act as synthetic agonists of PPARg and improve insulin sensitivity in part through stimulating adipogenesis in SAT [69, 70]. Thus, the critical pathophysiological factor in obesity-associated cardiometabolic risk is not the absolute amount of WAT but rather its capacity to carry out its critical role in regulating systemic lipid homeostasis.

The capacity for hyperplastic expansion in WAT is limited by the availability of adipocyte progenitors and their differentiation potential. The theory that adiposopathy in obesity is triggered by a deficiency in the capacity for adipogenesis is supported primarily by studies using cultured ASC isolated from obese humans or rodent models of obesity. A few studies have demonstrated impaired in vitro proliferation and differentiation capacity of ASC isolated from the stromal vascular fraction of obese humans [71, 72]. In a mouse model of diet-induced obesity, reversing the impairment in differentiation potential of ASC from inguinal SAT prevented the development of insulin resistance and hepatic lipid accumulation [73]. An older study showing reduced preadipocytes in the stromal vascular fraction of obese patients is often cited as evidence for impaired differentiation capacity in obesity [74]. However, in this study, preadipocytes in the stromal vascular fraction were identified solely on the basis of staining for the fatty acid binding protein, aP2 [74, 75]. More recent single cell RNAseq studies have lent support to the adipose tissue expandability hypothesis by showing diet-induced obesity in mice to cause shifts toward less differentiated and pro-inflammatory subpopulations of adipose progenitors [76, 77].

Another line of evidence supporting a role for impaired adipogenesis as a mechanism underlying adiposopathy comes from studies in aged humans and rodents. Aging is accompanied by a decrease in WAT volume and a redistribution of fat in favor of VAT depots. Thus, adiposopathy likely contributes to age-related increases in the risk for cardiometabolic disease. Several studies have demonstrated blunted differentiation capacity in ASC isolated from adipose depots of aged humans or rodents [78–80]. A study using single cell RNAseq in the SAT of mice showed that aging leads to depletion in populations of mesenchymal stem cells, committed ASC and preadipocytes [81]. Cellular senescence in aging is a proposed mechanism for the exhaustion of adipocyte progenitors and their blunted differentiation capacity. Interestingly, Li et al. showed premature senescence in freshly isolated adipocytes from insulin resistant obese humans [45], and other groups have observed markers of senescence in cultures of ASC isolated from obese rodents and humans [79, 82, 83]. Therefore, in both obesity and aging, cellular senescence may be either a cause or consequence of chronic inflammation in WAT, a hallmark of adiposopathy. Findings from our laboratory show that the effect of ROS on adipogenesis is non-linear, such that low levels of ROS potentiate adipogenesis, while high levels impair differentiation [51]. Thus, signals emanating from an adipose microenvironment that has progressed to a state of chronic inflammation and redox imbalance may instigate the transition from adaptive WAT remodeling to adiposopathy. Adipose tissue inflammation in obesity is not only attributable to excessively hypertrophied adipocytes but also to an increase in the presence of macrophages due to proliferation of resident macrophages and infiltration of monocytes, as well as a polarization toward the pro-inflammatory M2 subtype [84, 85]. Exposure of ASC to media enriched with M2 macrophages inhibits their differentiation [86]. Recent studies using single cell RNAseq reveal heterogeneity within the APC pool with subpopulations differing in proliferative and adipogenic potential [77, 87, 88]. Pro-inflammatory, anti-adipogenic ASC have been found to appear in higher abundance in SAT of aged mice [81, 88, 89] Interestingly, subpopulations of ASC give rise to adipocytes that differ in metabolic properties such as lipolytic activity and fatty acid uptake [87]. Therefore, phenotypic changes in the progenitor pool may negatively influence capacity for differentiation and self-renewal as well as adipocyte function. A study by Tang et al. demonstrated that prolonged treatment of mice with TZD resulted in a reduced differentiation capacity of isolated ASC and a decrease in the population of ASC without a change in the rate of apoptosis [90]. This finding suggests that the progenitor pool and its ability to self-renew may be finite. More studies are required to better understand the molecular and pathophysiological mechanisms responsible for reduced WAT plasticity and its role in the development of adiposopathy.

### Developmental programming of adiposity and wat plasticity

#### Establishment of the progenitor pool during fetal life

Establishment of WAT depots and their resident progenitor pools required for regulating lipid homeostasis throughout life occurs during critical developmental windows. In the human fetus, a high rate of growth in the last months of gestation coincides with a rapid increase in the accumulation of fat, primarily in SAT depots [91]. The human neonate is born with a high proportion fat mass relative to other mammals. In mice, rapid WAT expansion between birth and postnatal day 16 (P16) coincides with a 10–20% daily increase in body mass [92]. After the second phase of adipose tissue growth initiated at puberty onset, adipose depots are fully established, and adipocyte numbers remain static thereafter [92]. The time course of adipogenesis and lineage dynamics during the developmental establishment of WAT depots have been characterized by transgenic techniques in adipocyte labeling and lineage tracing. Using the AdipoChaser mouse, an inducible adipocyte-tagging system, Wang et al. demonstrated that terminal differentiation of adipocytes in SAT is initiated between E14 and E18 and stabilizes shortly after birth, whereas perigonadal adipocytes arise primarily in postnatal life [93]. Likewise, the AdipoTrak system that indelibly labels progenitors expressing PPARγ, a master regulator of adipogenesis, revealed that specification of progenitors in SAT begins at E14.5 and is completed by P2, while specification in perigonadal VAT depot occurs between P4 and P10 [94]. After the perinatal period of adipocyte differentiation, there is a second period of ASC proliferation during puberty, suggesting that the ASC population specified in utero is activated to drive the pubertal wave of WAT expansion [79]. Since progenitor and adipocyte populations in SAT are established before birth, fetal life is a critical window that sets the trajectory for the rapid fat accumulation that occurs in the neonatal and pubertal periods of postnatal development. Thus, the setpoint for adiposity is programmed in utero.

**Fig. 2:**
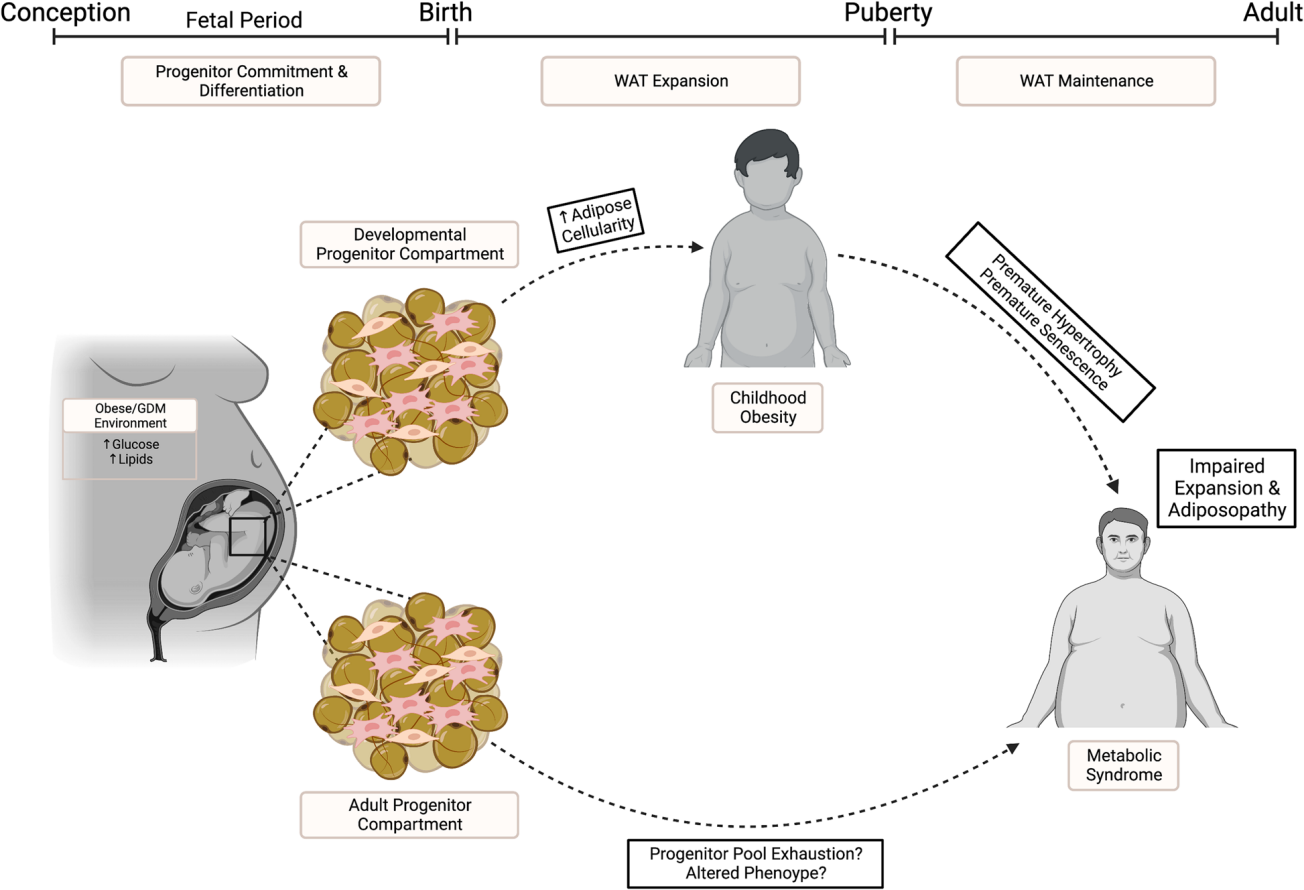
Working hypothesis of the role of the adipocyte progenitor pool in programming of MetS. Fetal life is a critical window of adipocyte progenitor commitment that establishes a compartment of developmental progenitors driving WAT expansion in early life as well as a distinct adult compartment. An intrauterine environment of overnutrition favors adipogenesis, accelerating the trajectory of WAT expansion that occurs in the perinatal and pubertal periods, thus raising the setpoint of adiposity and programming childhood obesity. Early life obesity may predispose to premature hypertrophic dysfunction and senescence, leading to impaired WAT plasticity. A pro-adipogenic phenotype of progenitors programmed in utero may also prematurely exhaust the progenitor pool, predisposing to adiposopathy and MetS

The adipocyte progenitor pool occupying established adult depots is not a residual population stemming from developmental adipogenesis but rather a distinct compartment with unique anatomical, functional, and regulatory characteristics. Developmental progenitors give rise to adipocytes during the establishment of WAT depots in early life, while adult progenitors serve as a reservoir for replacing adipocytes during normal cellular turnover and generating new adipocytes in response to increased lipid storage demands [55, 93, 94]. Using an adipocyte fate mapping model, Jiang et al. showed that progenitors giving rise to adipocytes in adult depots reside in a perivascular niche and start to appear at P20, while developmental progenitors are not localized to blood vessels. Deleting PPARγ from the perivascular lineage had no impact on WAT depots before P10 [94]. In agreement, another group showed that newly differentiated adipocytes after high fat feeding in adult mice arise from a mural population of progenitors, which are more abundant in the female SAT compared to male SAT [95]. Adipocyte-specific knock out of C/EBPa or Akt2 interfered with WAT remodeling in adult depots but had no impact on WAT in the perinatal period [55, 93], suggesting that molecular pathways driving developmental adipogenesis are distinct from those regulating WAT expansion in established depots. Intriguingly, the study by Jiang et al. mentioned above revealed that lineage commitment of the progenitor population that gives rise to adipocytes in adult tissue occurs during fetal life, prior to specification of the developmental compartment [94]. These seminal findings suggest that childhood and adult adiposity are uniquely regulated and highlight fetal life as a critical window that establishes the setpoint for both childhood and adult adiposity and programs later-life WAT plasticity (Fig. 2).

#### Maternal metabolic disease perturbs the trajectory of WAT development

Since fetal life is a critical window of WAT development that establishes adipocyte number and progenitor populations, perturbations to the intrauterine milieu are likely to influence the risk for obesity and WAT plasticity. Decades of research into the developmental origins of health and disease (DOHaD) firmly establish that early windows of organ development shape later life chronic disease risk. It is well recognized that maternal obesity or diabetes during pregnancy increases the risk for obesity and MetS in the offspring [13, 15, 16, 29, 96, 97]. The most common perinatal outcome associated with pregnancies complicated by obesity or diabetes is fetal macrosomia primarily due to elevated adiposity. Therefore, maternal metabolic disorders create an intrauterine metabolic milieu that perturbs the trajectory of WAT development, which likely plays a major role in programming of MetS.

According to the Pederson hypothesis, macrosomia is a result of transplacental transport of glucose under conditions of maternal hyperglycemia, leading to hypersecretion of insulin from the fetal pancreas. Indeed, there is an abundance of evidence supporting a critical role for maternal hyperglycemia, including from the Hyperglycemia and Adverse Pregnancy Outcome (HPAO) study which showed a linear relationship between maternal glucose and birth weight [98]. However, it is now recognized that other factors such as maternal adiposity and lipid metabolism play a role in promoting excess fetal adiposity [17, 19, 99, 100]. A study by Alfadhli et al. showed that GDM and obesity each independently increase the risk for fetal macrosomia, with obesity alone resulting in a higher birth weight than GDM alone and the greatest risk found when GDM and obesity were comorbid [18]. A study by Modi et al. investigated the relationship between maternal BMI and offspring adiposity in 105 mother-infant pairs approximately 2 weeks after delivery. These authors revealed positive associations between maternal BMI and WAT volume in newborns, assessed by whole-body MR imaging. Additionally, these authors determined that an incremental increase in maternal BMI results in an approximate 8 mL increase in WAT volume, demonstrating a strong, direct relationship between maternal body composition and developmental WAT expansion [17]. Similarly, Mitanchez et al. (2017) found that among nearly 500 mother-infant pairs, a BMI >/= 30 kg/m^2^ resulted in no difference in birthweight but increased skinfold thickness. Additionally, these authors showed elevated cord leptin in obese pregnancies, with both skinfold thickness and maternal leptin being positively associated with maternal weight gain during pregnancy [19]. Therefore, maternal body composition assessed directly through anthropomorphic assessment and indirectly by serum leptin levels predicts neonatal adiposity. Overall, there is evidence to support a role for both maternal adiposity and glucose levels in promoting excess fat accumulation in the fetus.

As maternal obesity or diabetes accelerate the accumulation of fat in fetal WAT depots, it follows that the intrauterine milieu in these pregnancies direct a greater proportion of stem cells towards the adipocyte lineage or terminal differentiation. However, the physiological cues that drive adipogenesis during development of WAT remain unresolved. Current understanding of the complex transcriptional pathways that govern adipocyte differentiation largely derives from in vitro models. Insulin, a potent adipogenic hormone that induces differentiation in ASC culture, increases in late gestation and is a major driver fetal growth. Therefore, fetal hyperinsulinemia is a likely candidate for mediating the excessive accumulation of WAT characteristic of macrosomic infants born to GDM or obese pregnancies. A study in sheep [101] showed that consumption of a high calorie diet by the ewe during pregnancy resulted in higher circulating glucose and plasma in the late gestation fetus, as well as increased adipocyte size and PPARγ expression in the perirenal WAT depot, the latter positively correlated with plasma glucose. An increase in adipocyte size along with high fat mass have also been observed in early postnatal life in rodents born to dams with diet-induced obesity. Since rodents are postnatal developers and born with very little fat, these findings suggest that overnutrition during the in utero window of progenitor commitment and differentiation is sufficient to program changes in adipose cellularity. Maternal overnutrition exclusively during the lactation period in rodents has been shown to independently program a propensity towards accelerated growth and fat mass accumulation and has an additive effect when combined with prenatal exposure [102–106]. Therefore, overnutrition during fetal and early postnatal life accelerate WAT development and thereby program a lifelong risk for obesity and MetS. However, it should be noted that the development of metabolic dysfunction in offspring born to obese or diabetic mothers is multifactorial, involving non-adipose mechanisms such as programming of hypothalamic circuits regulating appetite and satiety [105, 107–109].

Changes in adipocyte size in WAT depots suggest an acceleration in the maturation of WAT but do not dissect the relative contributions of progenitor proliferation, differentiation, and lipid deposition. In rodent models, changes in the expression of key regulators of differentiation in embryos or whole WAT lysates have been reported in weaning offspring born to high-fat fed dams [110–112]. However, it is difficult to interpret gene expression changes in whole WAT lysates as the expression of specific markers varies according to the stage of differentiation among a hierarchical population of progenitors, while many play a role in lipid or glucose metabolism in mature adipocytes. Several studies have more directly studied adipogenesis by using in vitro techniques to assess cell-intrinsic properties of adipose progenitors. Yang et al. (2013) showed that while there was no effect of maternal high-fat diet on embryonic weight, adipocyte progenitors isolated from embryonic fibroblasts on E14.5 exhibited greater differentiation capacity [112]. High expression of ZFP423, an early regulator of stem cell commitment to the adipocyte lineage, concurred with hypomethylation of the ZFP423 promoter in embryonic tissue of obese dams. In another study, the same group showed that at Pd21, in vitro differentiation potential was higher in progenitors isolated from epididymal VAT; however, after high fat feeding, adipocyte expansion rate and progenitor density were lower in offspring born to obese dams, suggesting that premature adipogenesis during perinatal development limited WAT plasticity in later life [113]. Using a rat model of maternal diet-induced obesity, Borengasser et al. (2013) reported higher differentiation capacity in stromal vascular cells isolated from retroperitoneal VAT in offspring of obese dams, which persisted at P100 [110]. Our laboratory used a mouse model of high pre-pregnancy maternal adiposity and revealed increased differentiation capacity of adipocyte progenitors isolated from inguinal SAT on Pd21 [114]. Boyle et al. studied mesenchymal stem cells from the umbilical cord of obese or lean mothers and found that cells isolated from obese pregnancies had elevated PPARg expression and increased differentiation potential [115]. Together, these findings demonstrate that maternal obesity influences cell-autonomous properties of adipocyte progenitors during early establishment of WAT depots but do not necessarily capture the developmental dynamics of stem cells preserved in their native microenvironment.

#### Programming of adiposity and WAT plasticity by maternal metabolic disease

An intrauterine milieu of overnutrition accelerates adipose maturation during the window of progenitor specification, commitment, and differentiation that establishes the cellularity of WAT depots. How this aberration in the developmental trajectory of WAT influences the progenitor population and WAT plasticity in later life remains unresolved. Does a greater endowment of the adult progenitor compartment raise the setpoint for adult adiposity and underlie the relationship between maternal obesity and offspring obesity? If a larger number of progenitors are directed towards terminal differentiation, does this limit the reservoir available for adaptation to changes in energy balance or predispose to premature senescence? Since excessive neonatal adiposity is the major outcome of pregnancies complicated by maternal metabolic disease, it is likely that fetal programming of WAT function and cellular composition is a key pathophysiological event in the development of MetS later in life.

It is well established that maternal obesity and diabetes are predictors of MetS in the offspring and its early onset. A study in the Pima, an indigenous group with the highest rate of T2DM in the world showed that hemoglobin A1c and systolic blood pressure were higher in children born to mothers that were diabetic during pregnancy compared to those born to mothers who developed T2DM after pregnancy, demonstrating the in utero environment to be an independent predictor of MetS [13]. Boney et al. reported 50% of children born large-for-gestational (LGA) age from a GDM pregnancy met the criteria for MetS at ages 6–11, compared to 30% of children with normal birth weight. There was also higher risk for MetS in children born LGA from non-diabetic pregnancies, suggesting macrosomia to be an independent risk factor [116]. A retrospective study reported a higher tendency of macrosomic babies to be obese at 3 years of age, and another study showed higher risk of obesity at 4 years of age in offspring born to mothers who were obese during pregnancy [117]. These findings suggest that fetal development in an intrauterine environment of nutrient-overabundance raises the setpoint for adiposity before puberty. A recent meta-analysis of 34 cohorts of mother-infant pairs revealed that the risk of offspring obesity after GDM was almost entirely accounted for by maternal BMI [118], suggesting that maternal adiposity plays a dominant role in transmitting obesity to the offspring. In contrast, the results from the Danish National Birth Cohort showed maternal fasting plasma glucose to be the strongest predictor of early-onset obesity in children born to mothers with GDM [119]. However, in this study, most women diagnosed with GDM were overweight or obese. In summary, programming of MetS in offspring exposed to maternal obesity or hyperglycemia appears to accelerate the trajectory of fat accumulation during the pre-pubertal period of WAT expansion.

It is possible that a perturbation in fetal WAT development not only programs a susceptibility to obesity, but limits WAT plasticity, thereby predisposing to early onset adiposopathy. Very few investigations have explored the role of WAT dysfunction as a mechanism linking macrosomia to the development of MetS. A few studies in rodent models of diet-induced maternal obesity show insulin resistance and increased VAT volume in the offspring to be accompanied by a higher degree of adipocyte hypertrophy and increased total cell number, suggesting an exaggerated adipogenic response in VAT [113, 120]. A study in our laboratory focused on inguinal SAT, as SAT is the body’s metabolic sink that expands under obesogenic conditions to protect against spillover of lipids in the secondary visceral depots and ectopic organs. Our results show that the shift in adipocyte size distribution in SAT of weaning pups born to dams with high adiposity persisted into adulthood and limited adipocyte expansion in response to high fat feeding. Limited SAT expansion coincided with hallmarks of adiposopathy such as impaired lipolysis in response to insulin [114]. Our study as well as others show that male offspring of adverse pregnancies are more susceptible to diet-induced insulin resistance. Interestingly, our data show that despite a similar degree of obesity, females from control pregnancies maintain lipid and glucose homeostasis after high fat/fructose feeding [114]. Thus, higher plasticity of SAT in females may be responsible sex differences in programming of MetS. Work from our lab [114] and other groups demonstrate that while male and female offspring of metabolically compromised pregnancies are similarly predisposed to the development of obesity, females tend to be protected from the cardiometabolic consequences of obesity [103, 111]. These sex differences are likely attributable to multiple mechanisms; however, sexual dimorphism in adipogenic responses and lipid buffering capacity may play a role. Further investigation is required to determine whether SAT expandability is impaired in offspring born to metabolically complicated pregnancies, as cell diameter does not necessarily reflect adipocyte age. This question can be more directly addressed by leveraging transgenic models of adipocyte labeling and new techniques in the identification and characterization of adipocyte progenitor populations.

## Conclusions

The weight of the obesity burden is escalating in low-income countries and among the pediatric population and is a significant global health problem that adds pressure to national health care systems. It is well established that maternal obesity and diabetes, which are now common complications of pregnancy, increase the risk for development of obesity and MetS in the offspring, often before adulthood. Herein, we argue that since the most common outcome of maternal obesity or GDM is excess fat mass accumulation during the fetal window of adipocyte progenitor commitment and differentiation, predisposition to childhood obesity and MetS in the offspring is programmed by accelerated adipogenesis in utero. Very few groups have pursued this hypothesis; however, several studies show a pro-adipogenic phenotype in ASC isolated from embryos or weaned pups from obese pregnancies. Although these results have not been validated by in vivo techniques, they are supported by shifts in cell size distribution toward larger adipocytes in WAT depots. Directing a greater number of stem cells toward the adipocyte lineage and terminal differentiation may accelerate the trajectory of WAT expansion during the perinatal and pubertal periods of adipose development and predispose to excessive hypertrophy, dysfunction, and senescence. Further, since the adult compartment of adipocyte progenitors are also established before birth, it is possible that an abnormal intrauterine metabolic milieu influences the endowment or phenotype of this population, which plays a critical role in maintaining systemic lipid homeostasis. Deficiencies in differentiation capacity of the adipocyte progenitor pool in established depots precipitates adiposopathy and the onset of obesity-associated MetS. Further research is required to elucidate the impact of perturbed WAT development as a mechanism of developmental programming.

## Data Availability

Not applicable.

## Abbreviations

Akt2: RAC-beta serine/threonine-protein kinase
ASC: Adipose stem cells
BMI: Body mass index
BrdU: Bromodeoxyuridine
CDC: Center for Disease Control
CEBPa: CCAAT/enhancer-binding protein alpha
COVID-19: Coronavirus disease 2019
CVD: Cardiovascular disease
DOHaD: Developmental Origins of Health and Disease
GDM: Gestational diabetes mellitus
MetS: Metabolic syndrome
MHO: Metabolically healthy obesity
PPARg: Peroxisome proliferator activated receptor gamma
RNAseq: Ribonucleic acid sequencing
ROS: Reactive oxygen species
SAT: Subcutaneous adipose tissue
T2DM: Type-II diabetes mellitus
TZD: Thiazolidinediones
US: United States of America
VAT: Visceral adipose tissue
WAT: White adipose tissue
ZFP423: Zinc-finger protein 423
